# Osteosynthesis versus endoprosthesis for the treatment of femoral neck fracture in Asian elderly patients

**DOI:** 10.1186/s12891-016-1123-7

**Published:** 2016-07-05

**Authors:** Joon Soon Kang, Yoon Sang Jeon, Chi Hoon Ahn, Tae Hoon Roh

**Affiliations:** Department of Orthopaedic Surgery, Inha University Hospital 7-206, 3-Ga Sinheung-dong, Jung-gu Incheon, 400-711 Korea

**Keywords:** Femoral neck fracture, Endoprosthesis, Bipolar hemiarthroplasty, Internal fixation, Complication, Osteoporosis

## Abstract

**Background:**

The purpose of this study was to compare the clinical results between osteosynthesis and endoprosthesis for femoral neck fractures in asian elderly patients, and to analysis the factors that may affect the failure of osteosynthesis.

**Methods:**

A retrospective review of 382 hips over 65-year old with femoral neck fracture was done. Within non-displaced fracture group, 81 cases (56.6 %) underwent internal fixation (IF) and with 62 cases (43.3 %) having bipolar hemiarthroplasty (BPHA). As for displaced fracture group, 60 cases (25.1 %) underwent internal fixation (IF) with 179 cases (74.8 %) having BPHA. Average follow-up period for the patients was 36.8 months. Analysis was conducted on complications depending on fracture types and osteoporosis, and clinical evaluation was done on gait capability by using Koval walking ability.

**Results:**

In non-displaced group, BPHA group showed statistically significant lower percentage of complications compared to IF group, but re-operation rate and the degradations of Koval score were no significant differences. In displaced group, complication, re-operation rate and the degradations of Koval score of BPHA group were statistically better than those of IF group. Association between osteoporosis and non-union is no statistically significant.

**Conclusions:**

Endoprosthetic replacement could be a primary option for displaced femoral neck fracture in elderly asian patients. The choice of surgical treatment methods of non-displaced fracture in elderly asian patients should be determined carefully considering the age and the presence of osteoporosis.

## Background

The incidence of eldely femoral neck fracture has been rising every year due to increase in the average life span with recent development of medical technology [1]. Especially in Asia, the incidence of this fracture is steadily increasing and in 2050, more than 50 percent of hip fracture is expected to occur in Asia [2]. Arthroplasty and internal fixation can be considered as operative treatments for femoral neck fracture. In young patients, internal fixation should be tried even in severe displaced femoral neck fractures . On the other hand, it has been controversial for aged patient. The failure rate of osteosynthesis is reported to be as high as 20 % in elderly femoral neck fracture due to osteonecrosis, nonunion and fixation failure, and also post-operative ambulation might be delayed in this population due to the difficulty of achieving firm fixation [3, 4]. Especially, the elderly asian women have low bone density compared to the westerners, which may be a risk factor that can lead to osteoporotic fractures and show the higher possibility of fixation failure after femoral neck fracture [5]. The purpose of this study was to compare the clinical results between osteosynthesis and endoprosthesis for femoral neck fractures in asian elderly patients, and to analysis the factors that may affect the failure of osteosynthesis.

## Methods

This study included 382 patients over 65 years old who were diagnosed with femoral neck fracture during the period from January 1996 to January 2013. Average follow-up period for the patients was 36.8 months (range: 24 ~ 148 months). Out of these cases, internal fixation (IF) with cannulated screw was done for 141 cases, and bipolar hemiarthroplasty (BPHA) was performed for 241 cases. Within non-displaced fracture group, 81 cases (56.6 %) underwent internal fixation (IF) and with 62 cases (43.3 %) having bipolar hemiarthroplasty (BPHA). As for displaced fracture group, 60 cases (25.1 %) underwent internal fixation (IF) with 179 cases (74.8 %) having BPHA. Patients who have medical history (angiopathy, neoplasia, pathologic fracture, rheumatoid arthritis, osteomyelitis, and those who take steroids) that may possibly affect any incidence of femoral neck avascular necrosis and fixation failure were excluded. Diagnosis of osteoporosis was defined as those whose bone mineral density value after dual-energy x-ray absorptiometry (DEXA) was lower than −2.5 based on the minimum T-score. In case DEXA was not done, it was defined as Singh index grade 4 or lower based on trabecular type in the femoral head and proximal femoral. Clinical evaluation was made on gait capability with Koval walking ability. Failure of internal fixation was defined as non-union and avascular necrosis, and failed BPHA was defined as twice or more recurrent dislocation, aseptic loosening, periprosthetic fracture and infection.

The degree of reduction was assessed based on the Garden alignment index, which was measured by using anterior-posterior (AP), and lateral plain radiographs of the hip joint immediately after operation. The baseline was set such that Garden alignment index was between 160 and 180 degrees in the AP radiograph and between 170 and 190 degrees in the lateral radiograph. If both radiographs fell within the range, it was assessed as excellent. If only one item fell within the range, it was defined as good. Finally, if none fell in the range, it was poor. Two indicators were used to evaluate the quality of firm fixation. First, in the AP, and lateral plain radiographs, the average distance between the screw tip and the subchondral boundary of femoral head was measured. Second, using the same radiographs, the fixation at three-points was assessed through the inferotemporal cortical bone of the femoral neck. The case was defined as excellent if the average distance to subchondral boundary of femoral head was 10 mm or shorter and the fixation at 3-points was satisfactory. With the distance longer than 10 mm and satisfactory fixation at the 3- points, or average distance of 10 mm or shorter and unsatisfactory fixation at the 3 points, the case was defined as good. If the average distance was 10 mm or longer and there was unsatisfactory fixation, it was assessed as poor.

To evaluate avascular necrosis, the Ficat classification were used. Patients with suspected avascular necrosis were checked using either radiographs or radiographs and MRI [6]. Failure in fixation was diagnosed when bone union was not observed in radiography or there was displacement of fixation area even three months after operation with continued pain in hip joint.

Internal fixation was done, with spinal anesthesia for all patients, followed by anatomical reduction around fractured area under C-arm guidance and with three to four 6.5 mm cannulated screws. All patients were allowed to toe touch ambulation and non-weight bearing excercies under a physiotherapist assistant at least for six weeks after operation, and during hospitalization, same rehabilitation therapy program was provided.

For BPHA, posterolateral approach was done after placing a patient into lateral recumbent position. Cemened femoral stem is inserted in only 4 hips. Cementless femoral stem (Zweymuller stem, Anatomoc medullary stem and Coren stem) were insert in most of cases. Until postoperative sixth week, partial weight-bearing gait was done by using walker or crutches, and afterwards free gait was allowed. Follow-up observation as out-patient was done in six weeks, three months, six months, and one year interval after operation, and then with annual follow-up. Statistical validation was conducted through using paired *t*-test and chi-square analysis on each indicator, by using IBM SPSS 19.0, and *p* value less than 0.05 was deemed significant.

## Results

In non-displaced group, Average age at time of injury of IF group was 73.1 years and BPHA group was 77.2 years In terms of gender, 27 male and 54 female were included in the IF group, while there were 18 male and 44 female in the BPHA group. American Society of Anesthesiologists Grade (ASA) was 2.55 for IF group and 2.78 for BPHA group. Average time required from injury to operation was 2.6 days for IF group and 3.12 days for BPHA group. and BMD was −3.5 for IF group and −5.25 for BPHA group. In aspects of age & BMD, there was statistically significant difference between the two groups.

In displaced group, average age at time of injury of IF group was 74.3 years and BPHA group was 75.3 years In terms of gender, 21 male and 39 female were included in the IF group, while there were 50 male and 129 female in the BPHA group. ASA was 2.75 for IF group and 2.75 for BPHA group. Average time required from injury to operation was 3.08 days for IF group and 2.83 days for BPHA group. and BMD was −4.13 for IF group and −4.92 for BPHA group. In only aspect of BMD, there was statistically significant difference between the two groups [Table 1].

**Table 1:** Patient demographics

	Non-displaced (*n* = 143)			Displaced (*n* = 239)		
	IF (*n* = 81)	BPHA (*n* = 62)	*P* value	IF (*n* = 60)	BPHA (*n* = 179)	*P* value
Age	73.1	77.2	**0.001**	74.3	75.3	0.361
M/F	27/54	18/44	0.583	21/39	50/129	0.299
ASA	2.55	2.78	0.13	2.75	2.75	0.24
Timing of surgery (day)	2.6	3.12	0.281	3.08	2.83	0.915
BMD	−3.5	−5.25	<**0.001**	−4.13	−4.92	0.019

Statistically significant results are indicated in bold

*P*-value for difference between IF and BPHA

Looking at mortality rate after operation, In non-displaced group, IF group lost 1 patient (1.2 %) in the first year after operation and 4 patients (4.9 %) in the second year, while BPHA group lost 3 patients (4.8 %) and 7 patients (11.3 %) in the first and second year. There was no significant difference between the two groups (*p* = 0.15).

In displaced group, IF group lost 7 patient (11.6 %) in the first year after operation and 15 patients (25 %) in the second year, while BPHA group lost 3 patients (4.8 %) and 7 patients (11.3 %) in the first and second year, respectively. but there was no significant difference between the two groups (*p* = 0.19).

As for Koval score for clinical assessment, in non-displaced group, IF group showed change of 1.3 from 1.5 before operation to 2.8 during the last follow-up, while BPHA group recorded change of 1.07 from 1.54 to 2.61 during final follow-up. Though BPHA group showed lower reduction in gait decrease, it was not statistically significant difference (*p* = 0.093).

In displaced group, IF group showed change of 1.35 from 1.58 before operation to 2.93 during the last follow-up, while BPHA group recorded change of 1.01 from 1.62 to 2.63 during final follow-up. The degradations of Koval score of BPHA group were statistically better than those of IF group (*p* = 0.014).

As for complications, in non-displaced group, IF group had 6 patients (7.4 %) with avascular necrosis and 4 patients (4.9 %) with non-union, leading to re-operation of 19 patients (6.1 %), and BPHA group had 1 patient (1.6 %) with infection, leading to re-operation of 1 patient (1.6 %). BPHA group showed statistically significant lower percentage of complications compared to IF group, (*p* = 0.017) but re-operation rate was no significant differences (*p* = 0.17).

In displaced group, IF group had 17 patients (28.3 %) with avacular necrosis and 7 patients (11.6 %) with non-union, leading to re-operation of 14 patients (23.3 %), while BPHA group had 4 patienst (2.2 %) with dislocation and 2 patient (1.1 %) with periprosthetic fracture, leading to re-operation of 5 patient (2.7 %). BPHA group showed statistically significant lower percentage of both complications and re-operations compared to IF group (*p* < 0.001).

Looking at incidence rate of complications depending on BMD among patients with internal fixation, non-displaced groups showed higher incidence of complications when they had osteoporosis. However, it was not statistically significant [Table 2].

**Table 2:** Clinical outcome of internal fixation in osteopenic & osteoporotic patients

	Non-displaced (*n* = 81)		Displaced (*n* = 60)	
	BMD > −2.5	BMD < −2.5	BMD > −2.5	BMD < −2.5
	(*n* = 27)	(*n* = 54)	(*n* = 17)	(*n* = 43)
Fixation failure	1 (3.7 %)	3 (5.5 %)	2 (11.7 %)	5 (11.6 %)
*P* value	0.45		0.60	

Garden alignment index, which was measured based on AP plain radiography of hip joint immediately after operation, was average 166.4 degrees, with 178 degrees on average from lateral radiography. Average distance between screw tip and subchondral boundary of femoral head was 8.1 mm. As for anatomical reduction after operation, there were 110 excellent cases, with 24 good and 7 poor cases. When the level of reduction was lower, incidence of avascular necrosis increased, but it was not statistically significant (*p* = 0.154). With poor level of reduction after operation, incidence of fixation failure increased and it was statistically significant (*p* = 0.005). [Table 3] Similarly, with poor level of fixation, incidence of avascular necrosis increased but it was not statistically significant (*p* = 0.179). As the quality of firm fixation was worse, fixation failure increased to statistically significant level (*p* = 0.013) [Table 4].

**Table 3:** Quality of reduction

Number	Excellent	Good	Poor	*P*-value
(%)	(*n* = 110)	(*n* = 24)	(*n* = 7)	
AVN group	13 (11.8 %)	6 (25 %)	4 (57.1 %)	*P* = 0.154
Fixation failure group	3 (2.7 %)	5 (20.8 %)	3 (42.8 %)	*P* = 0.005
No complication group	94 (85.4 %)	13 (54.1 %)	0 (0 %)

**Table 4:** Quality of firm fixation

Number	Excellent	Good	Poor	*P*-value
(%)	(*n* = 48)	(*n* = 66)	(*n* = 27)	
AVN group	4 (8.3 %)	8 ( 12.1 %)	11 (40.7 %)	*P* = 0.179
Fixation failure group	1 (2 %)	3 (4.5 %)	7 (25.9 %)	*P* = 0.013
No complication group	43 (89.5 %)	55 (83.3 %)	9 (33.3 %)	

## Discussion

Internal fixation is less invasive technique than arthroplasty and may be expected to reduce mortality rate. But in a recent meta-analysis study, there were no significant difference for mortality rate at mid-term or long-term follow up between IF and arthroplasty [7]. Similarly, our study did not have any statistically significant difference in its follow-up observation.

As for complications and re-operation rate, the results of a meta-analysis study has showed that arthroplasty is better than internal fixation in displaced fracture, which was similar to the outcome of this study [7]. Therefore BPHA could be considered as a preferred treatment for displaced fracture in elderly Asian patients. But, in non-displaced fracture, a recent study reported that healing complication rate of internal fixation group was about 15.3 %, so arthroplasty remains a controversial alternative to internal fixation [8]. Our study showed that in non-displaced group, IF group complication rate was 12.3 %, which was significant higher than BPHA group, but re-operation rate were no significant differences. Thus, in elderly asian patients, the results of non-displaced group was not significantly different from that in the western.

In aspects of age & BMD, there was statistically significant difference between IF and BPHA in non-displaced groups. The age as the predictor affecting the risk of nonunion has been well known already [9]. But the effect of osteoporosis on internal fixation for femoral neck fracture has been still under controversy [10]. BMD could significantly impact on cut-out holding power of screw equipment [11]. When BMD is lower than 4.0 g/cm^2^, fixation ability would be poor and arthroplasty would be better choice [12]. In addition, even in the case of non-displaced fractures, patients with severe osteoporosis have showed a relatively high incidence of nonunion or fixation failure [13]. Even though non-displaced fractures had a better blood supply and bony contact than displaced fractures, the stability of internal fixation may be affected by osteoporosis as a risk factor of fixation failure [13, 14]. Furthermore, one study reported that the BMD of elderly Asian women was generally lower than that of the westerners [5]. In this study, there was no statistical significance between presence of osteoporosis and fixation failures. However, we mostly performed BPHA considering fixation failure and nonunion in elderly osteoporotic patients, that might be affected to such results. Therefore, the surgeon should be ask to pay close attention to choose the treatment option even in case of non-displaced fracture in elderly asian patients.

There are numerous different opinions on risk factors causing complications of internal fixation. Displacement and insufficient reductions could be common cause of complications. According to a study, the period from injury to operation was not largely relevant as a factor causing complications of internal fixation, and insufficient reduction was a predictive factor for complications [15]. In addition, some reported that displaced neck fracture or poor reduction led to higher probability of non-union in using internal fixation [16]. Therefore, if acceptable reduction is not possible, arthroplasty should be considered with priority.

In the IF group, this study showed a result that In case anatomical reduction was well done, incidence rate of fixation failure was significantly lower than in the case of poor reduction, but incidence rate of femoral neck avascular necrosis was not significantly different. As the number of poor reduction cases was small in this study, it would be difficult to place statistical significance in numbers. Also, in most patients included in this study, anatomical reduction was well performed. Since accuracy of reduction has already been known as an important predictive factor, in potential cases that were expected to be difficult to achieve good anatomical reduction, arthroplasty was used from the beginning and that choice contributed to lower number of poor reduction cases.

Arthroplasty group had complications of such as recurrent dislocation and infections. In particular, dislocation is the major concern after total hip arthroplasty for the treatment of femur neck fractures [17, 18]. In a recent meta-analysis, THR in patients with fractures of the femoral has higher dislocation rates compared with hemiarthroplasty [19]. For this reason some authors do not recommend THR as the treatment of choice in elderly patients with a fracture of the femoral neck. We performed hemiarthroplasty in all hips and our data showed a relatively low dislocation rate.

As for clinical prognosis assessed with level of gait reduction, BPHA group in displace fracture showed statistically lesser decrease during the final follow-up. But a meta analysis study showed better result with arthroplasty in early progress observation, and during the final follow-up, there was no difference or reduction was observed [7]. Reason for this outcome would be due to the relatively short follow up period, therefore futher study will be required to explane this difference.

This study performed restricted weight bearing after IF and arthroplasty. Generally, young patients underwent aggressive rehabilitation. but elderly osteoporotic patients have showed a relatively high incidence of varus collapse, screw migration [20]. And in one study, it had been reported that Weight-bearing and non-weight-bearing exercise programs produce similar effects on strength, balance, gait and functional performance among inpatients soon after hip fracture [21]. So we performed protective rehabilitation, which was allowed to toe touch ambulation and non weight bearing excercies, under a physiotherapist assistant. And the results showed no difference with the other papers in terms of clinical outcomes. so we think this protective rehabilitation would not have given a great impact on the clinical outcome.

## Conclusions

In displaced fracture, IF group showed significant higher incidence of complications and re-operation. Compared with IF, hemiarthroplasty can reduce the need for surgical revisions, decrease the incidence of complications and show relatively good results in terms of early functional outcome and mortality. Therefore, hemiarthroplasty could be considered as a preferred treatment for displaced fracture in elderly asian patients. The choice of surgical treatment methods of non-displaced fracture in elderly asian patients should be determined carefully considering the age and the presence of osteoporosis, further well planned study relevant to association between osteoporosis and fixation failure will be needed.

## Abbreviations

BMD, bone mineral density; BPHA, bipolar hemiarthroplasty; IF, internal fixation; THR, total hip replacement
